# Dissecting Japan's Dengue Outbreak in 2014

**DOI:** 10.4269/ajtmh.15-0468

**Published:** 2016-02-03

**Authors:** Mikkel B. Quam, October Sessions, Uma Sangumathi Kamaraj, Joacim Rocklöv, Annelies Wilder-Smith

**Affiliations:** Epidemiology and Global Health Unit, Department of Public Health and Clinical Medicine, Umeå University, Umeå, Sweden; Emerging Infectious Diseases Program, Duke–National University of Singapore Graduate Medical School, Singapore; Lee Kong Chian School of Medicine, Nanyang Technological University, Singapore

## Abstract

Despite Japan's temperate climate, a dengue outbreak occurred in Tokyo for the first time in over 70 years in 2014. We dissected this dengue outbreak based on phylogenetic analysis, travel interconnectivity, and environmental drivers for dengue epidemics. Comparing the available dengue virus 1 (DENV1) *E* gene sequence from this outbreak with 3,282 unique DENV1 sequences in National Center for Biotechnology Information suggested that the DENV might have been imported from China, Indonesia, Singapore, or Vietnam. With travelers arriving into Japan, Guangzhou (China) may have been the source of DENV introduction, given that Guangzhou also reported a large-scale dengue outbreak in 2014. Coinciding with the 2014 outbreak, Tokyo's climate conditions permitted the amplification of *Aedes* vectors and the annual peak of vectorial capacity. Given suitable vectors and climate conditions in addition to increasing interconnectivity with endemic areas of Asia, Tokyo's 2014 outbreak did not come as a surprise and may foretell more to come.

Endemic in at least 100 countries, dengue is currently regarded as world's most important mosquito-borne viral disease.^1^ Although most of the disease burden is limited to areas with tropical and subtropical climates, evidence suggests that temperate areas may be increasingly at risk as the geographic distribution of relevant vectors expands.^2^–^4^ Although Japan has a temperate climate, the first dengue outbreak in over 70 years occurred in 2014.^5^,^6^ The outbreak was due to dengue virus serotype 1 (DENV-1) and occurred in Tokyo, resulting in 160 dengue cases reported between August and October 2014.^5^,^6^ For an outbreak to occur in a previously dengue-free country, several factors must converge, such as importation of the DENV, presence of the vector, and favorable ecological and climatic conditions for the transmission of virus by vectors.

As Japan is geographically close to many countries in Asia that are highly endemic for dengue, it follows that the virus responsible for the 2014 dengue outbreak was likely imported from a country with high connectivity to Japan. Viral genetic information can assist in identifying the most likely country of importation,^2^ as can travel information.^2^,^7^ The probability of importation events occurring depends on the dengue activity in departure countries and the volume of travelers arriving.^2^,^7^

Vectorial capacity is the vector's ability to spread disease among humans and is dependent on vector density, mosquito-to-human biting rate, vector survival rate, and extrinsic incubation period; all of which are highly temperature-sensitive parameters.^4^ Relative vectorial capacity (rVc) has been proposed as an indicator for dengue epidemic potential.^4^ rVc for *Aedes* mosquitoes, the main vectors for DENVs, is thought to now have reached levels that are conducive for DENV transmission even in some temperate countries.^3^,^4^

We examined the factors that may have facilitated the dengue outbreak in Tokyo during 2014 by analyzing the available viral genetic information and data on travelers arriving in Japan from dengue-endemic Asian countries. We also calculated the rVc of *Aedes* mosquitoes in the months preceding the Tokyo outbreak. Finally, we assessed the seasonal rVc to explore the dengue epidemic potential in Japan.

Multiple sequence alignment of the available DENV-1 *E* gene sequence from the 2014 dengue outbreak in Tokyo and 3,282 unique DENV-1 sequences present in National Center for Biotechnology Information was carried out using a fast Fourier transformation method in MAFFT v6.940b (Kyoto, Japan).^8^ The approximately maximum likelihood phylogenetic tree was generated using generalized time-reversible model of nucleotide evolution in FastTree v2.1.7 (Berkeley, CA).^9^ FastTree uses SH-like local supports with 1,000 resamples to estimate and validate the reliability of each split in the tree. From the tree, the branch containing the sequence from the Japan 2014 outbreak (DENV-1/JP/Hu/Saitama/NIID100/2014, GI:686207807) was selected and a more robust maximum likelihood phylogenetic tree was created using RAXML with 100 bootstrap replications.^10^ Trees were visualized using FigTree v1.4.2 (Edinburgh, Scotland; http://tree.bio.ed.ac.uk/software/figtree/). Results of the phylogenetic analysis suggest a strong relationship between the Japan 2014 virus and a virus that was first identified in the Singapore dengue epidemic of 2005 (also seen in 2004–2006 and 2008–2009) and then circulated for a number of years in Asia: Vietnam (2008), Indonesia (2010, 2013), and China (2005, 2013) (Figure 1

**Figure 1.**
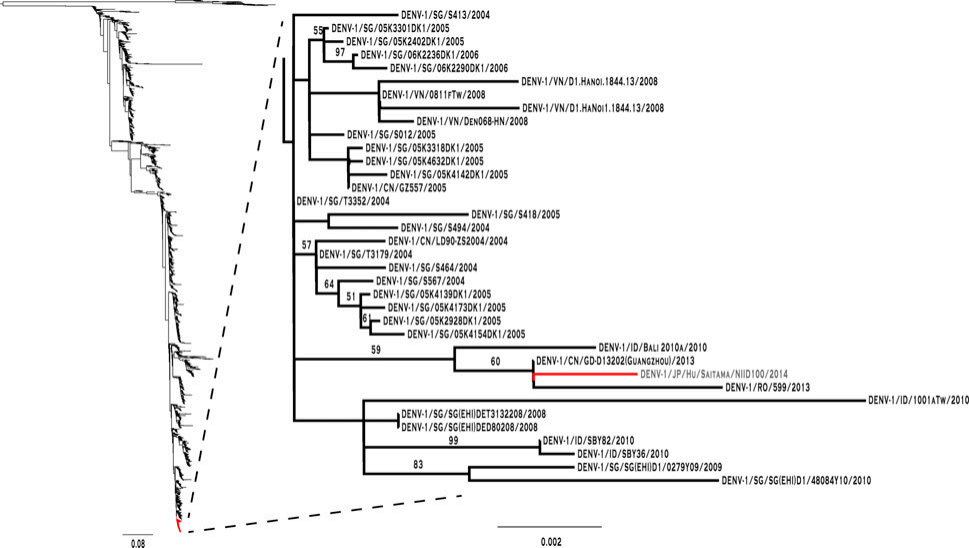
Phylogenetic tree of 2014 dengue index case. Phylogenetic tree for similarity of the strain of dengue virus serotype 1 (DENV-1) isolated from the 2014 outbreak in Tokyo, Japan, with other reported strains; the cases are labeled according to dengue viral serotype (DENV-1), country of isolation in International Organization for Standardization two-letter code (JP), laboratory identifiers of the isolate (Hu/Saitam/NIID100), and year of isolation (2014). The index case isolates from the outbreak in Tokyo are DENV-1/JP/Hu/Saitama/NIID100/2014 (marked in red) shown with neighboring sequences including DENV-1/CN/GD-D13202(Guangzhou)/2013 from China (CN) in 2013, DENV-1/RO/599/2013 imported to Romania (RO) from Indonesia (ID) in 2013, numerous strains from Singapore (SG) since 2004, and a few strains from Vietnam (VN) in 2008, among others. See also Supplemental Table 1 for GenBank Identifiers.

, Supplemental Table 1).

Furthermore, we obtained the Japan National Tourism Organization's data^11^ on inbound travelers between January and September 2014 from dengue-endemic countries in the southeast and east Asia arriving in Japan. Taiwan, China, and Hong Kong had by far the highest travel volume to Japan, followed by Malaysia and Singapore. Figure 2

**Figure 2.**
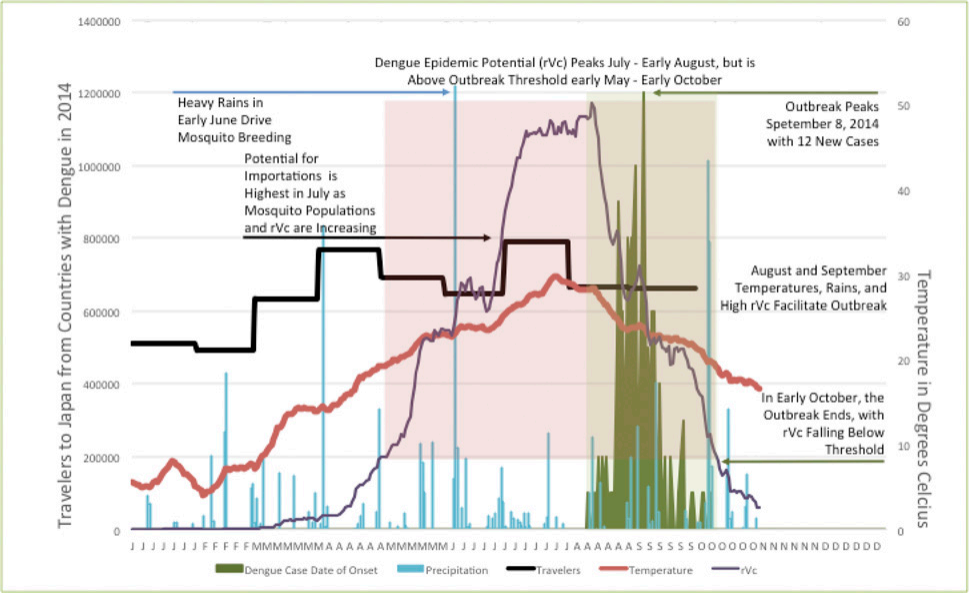
Conditions facilitating the 2014 outbreak of dengue in Tokyo, Japan. Red line: temperature (15-day moving average, degree Celsius, 4.0–29.8°C); blue lines: rainfall (daily millimeters of precipitation, blue bars, 0–244 mm/day); purple line: relative vectorial capacity (rVc), 0–1; and pink shaded box: dengue epidemic potential from May to early October in Tokyo, based on relative vectorial capacity being above threshold. Black line: reported incoming travelers from countries reporting dengue cases in 2014 arriving in Japan (monthly total, 492,300–790,046); green line: epidemiological curve of the 2014 outbreak of Tokyo; and green shaded box: period of the dengue outbreak in Tokyo (early August to early October).

shows that May–September coincides with the highest travel volume into Japan from all dengue-endemic countries combined.

Finally, we calculated the rVc for *Aedes* vectors to quantify the dengue epidemic potential based on temperature-dependent parameters. Our calculations followed a modified Ross–McDonald model:

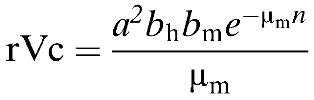
where *a* is the average daily vector biting rate, *b*_h_ the probability of vector to human transmission per bite, *b*_*m*_ the probability of human to vector infection per bite, *n* the duration of the extrinsic incubation period, and μ_m_ the vector mortality rate, all of which vary according to temperature.^4^ The temperature-dependent parameters for each of these variables needed to calculate rVc were derived from published experiments on *Aedes aegypti* according to methods described in detail previously, which included diurnal variation.^4^,^12^,^13^ We used the Ross–McDonald equation using the same temperature-dependent parameters for calculating rVc as given by Liu-Helmersson and others,^4^ but with updated temperature time-series datasets: the Met Office Integrated Data Archive System dataset^14^ for Tokyo and Climatic Research Unit (CRU) dataset^15^ for the time period leading up to the 2014 outbreak in Tokyo (Figure 2). In addition, we obtained the daily precipitation and mean temperatures for Tokyo,^14^ which were displayed alongside calculated rVc, the incoming travel volume from countries reporting dengue in 2014,^11^ and the epidemic curve of the 2014 dengue outbreak in Tokyo^5^ to show the temporal alignment of multiple factors (Figure 2). Figure 2 highlights the time window in 2014 that was most conducive for dengue epidemics in Tokyo. Taking into account travel volume, rVc, mean temperature, and precipitation, May–October was the most probable period for a dengue outbreak.

Using gridded (0.5° latitude × 0.5° longitude) climate data for the whole country of Japan (2004–2013) from the CRU 3.22 dataset,^15^ we also calculated decadal average rVc for Japan, which we aggregated and mapped by season (Figure 3

**Figure 3.**
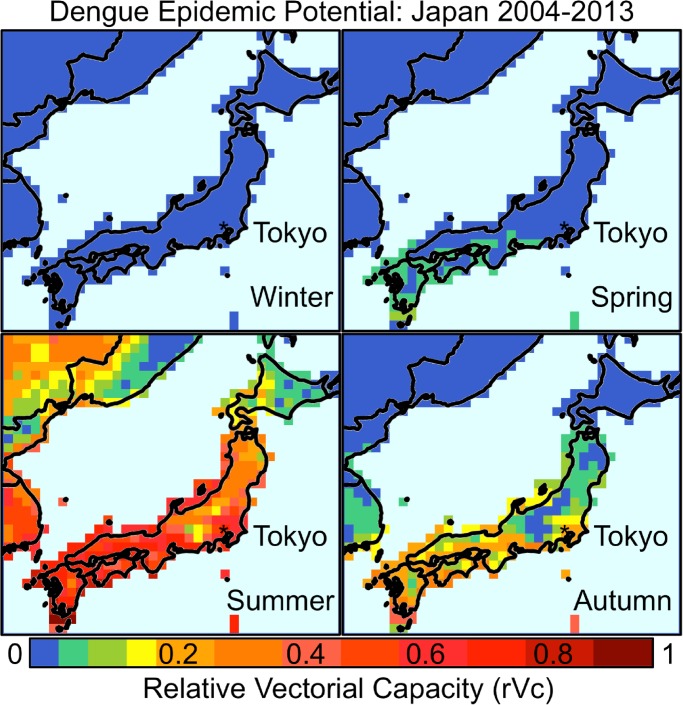
Seasonal mapping of dengue epidemic potential in Japan, 2004–2013. Relative vectorial capacity (rVc) ≥ 0.2 (orange-red) represent above threshold outbreak potential; rVc ≤ 0.2 (blue-yellow) represent below threshold outbreak potential; winter: December–February; spring: March–May, summer: June–August, and autumn: September–November.

). We found that rVc varied by season and geographical location in Japan. Figure 3 shows dengue epidemic potential as measured by rVc peaked during the summer months of June–August and was highest in southern Japan, while parts of northern Japan remained below the epidemic threshold. Despite the fact that we used rVc calculations based on *Ae. aegypti*, our results mirror previous findings^16^ of *Aedes albopictus*' seasonal activity and spatial extent in Japan and also correspond to the outbreak timing in 2014. A minor limitation of the rVc calculations is that the methods were originally developed based on *Ae. aegypti* not *Ae. albopictus*.^4^ As there is a lack of published data for *Ae. albopictus* on temperature-driven variations in the five parameters that constitute rVc, we had to rely on such data for *Ae. aegypti*. Although we would expect differences between the two vectors with regard to rVc, such differences are likely to be small, as a study on global temperature constraints of these two vectors points out.^3^

In conclusion, the phylogenetic similarity of DENV-1 *E* gene isolated from the 2014 outbreak in Japan with viruses from China, Indonesia, Singapore, and Vietnam renders any of these four countries a likely source of importation. Taking into account the high travel volume into Japan, Guangzhou could well have been the source of DENV introduction that triggered Tokyo's outbreak, given that Guangzhou had a large-scale dengue outbreak in 2014.^17^

Our findings show that several conducive factors converged preceding and during the time of the dengue outbreak in Tokyo, from August until October 2014. Climate conditions, particularly temperature and precipitation, were favorable for the amplification of *Aedes* vectors. Furthermore, the ability of the vector to transmit dengue, as measured by the rVc, was highest at the time of the 2014 outbreak. A previous global study showed small increasing trends for *Aedes* mosquitoes' vectorial capacity over the past century; and large increases are expected by the end of this century in temperate Northern Hemisphere regions using climate change projections.^4^ Therefore, despite Japan's temperate climate, dengue epidemic potential not only already exists but also is likely to increase over the next decades, exacerbated by the ongoing geographic expansion of *Aedes* vectors within Japan.^16^ Under scenarios of changing climate and increasing regional travel, these findings suggest that Japan will face more dengue outbreaks in the future. Considering the difficulties in controlling endemic and epidemic dengue activity in countries such as Singapore despite the expenditure of considerable resources, Japan should continue to be proactive about preventing and immediately mitigating future outbreaks. Enhanced vector surveillance and more stringent vector control measures may be necessary to preemptively combat this threat to ensure that dengue transmission in Japan does not become commonplace. However, the most important strategy to prevent dengue in Japan would be more effectively controlling dengue in all dengue-endemic countries, facilitated by an efficacious and safe vaccine, once available.

## Supplementary Material

Supplemental Table.
