# Supplementary material for: Dissecting Japan's Dengue Outbreak in 2014
Source: Am J Trop Med Hyg. 2016 Feb 3;94(2):409–12. doi: 10.4269/ajtmh.15-0468 (PMC4751952; doi:10.4269/ajtmh.15-0468)
Supplement: Supplementary file 1 [file SD6.pdf]

Supplemental Table 1

GenBank identifiers corresponding to Figure 1

Strain	GenBank ID
DENV-1/SG/S413/2004	GI:158343463
DENV-1/SG/05K3301DK1/2005	GI:158851745
DENV-1/SG/05K2402DK1/2005	GI:158851729
DENV-1/SG/06K2236DK1/2006	GI:158851829
DENV-1/SG/06K2290DK1/2006	GI:158851831
DENV-1/VN/D1.Hanoi.1844.13/2008	GI:558854699
DENV-1/VN/0811fTw/2008	GI:385718235
DENV-1/VN/D1.HaNoi1.1844.13/2008	GI:558854767
DENV-1/VN/Den068-HN/2008	GI:330339276
DENV-1/SG/S012/2005	GI:158343457
DENV-1/SG/05K3318DK1/2005	GI:158851747
DENV-1/SG/05K4632DK1/2005	GI:158851825
DENV-1/SG/05K4142DK1/2005	GI:158851783
DENV-1/CN/GZ557/2005	GI:409187395
DENV-1/SG/T3352/2004	GI:158343499
DENV-1/SG/S418/2005	GI:158343441
DENV-1/SG/S494/2004	GI:158343471
DENV-1/CN/LD90-ZS2004/2004	GI:381413870
DENV-1/SG/T3179/2004	GI:158343491
DENV-1/SG/S464/2004	GI:158343479
DENV-1/SG/S567/2004	GI:158343459
DENV-1/SG/05K4139DK1/2005	GI:158851779
DENV-1/SG/05K4173DK1/2005	GI:190889633
DENV-1/SG/05K2928DK1/2005	GI:158851739
DENV-1/SG/05K4154DK1/2005	GI:158851789
DENV-1/ID/Bali 2010a/2010	GI:396085274
DENV-1/CN/GD-D13202(Guangzhou)/2013	GI:636528351
DENV-1/JP/Hu/Saitama/NIID100/2014	GI:686207807
DENV-1/RO/599/2013	GI:584458393
DENV-1/ID/1001aTw/2010	GI:385718333
DENV-1/SG/SG(EHI)DET3132208/2008	GI:294986481
DENV-1/SG/SG(EHI)DED80208/2008	GI:294986425
DENV-1/ID/SBY82/2010	GI:353742412
DENV-1/ID/SBY36/2010	GI:353742390
DENV-1/SG/SG(EHI)D1/0279Y09/2009	GI:363499448
DENV-1/SG/SG(EHI)D1/48084Y10/2010	GI:363499472
